# The relationship between severity of liver steatosis and metabolic parameters in a sample of Iranian adults

**DOI:** 10.1186/s13104-020-05059-5

**Published:** 2020-04-16

**Authors:** Helda Tutunchi, Maryam Saghafi-Asl, Mohammad Asghari-Jafarabadi, Alireza Ostadrahimi

**Affiliations:** 1 Nutrition Research Center, Student Research Committee, School of Nutrition & Food Sciences, Tabriz University of Medical Sciences, Tabriz, Iran; 2 Nutrition Research Center, Department of Clinical Nutrition, School of Nutrition & Food Sciences, Tabriz University of Medical Sciences, Tabriz, 5166614711 Iran; 3 Road Traffic Injury Research Center, School of Health, Tabriz University of Medical Sciences, Tabriz, Iran

**Keywords:** Body mass index, Metabolic parameters, NAFLD, Obesity, Ultrasonography

## Abstract

**Objectives:**

This study aimed to examine the relationship between severity of liver steatosis and metabolic parameters in a sample of Iranian adults. In this cross-sectional study, a total of 95 subjects aged > 20 years newly diagnosed with NAFLD were recruited. NAFLD was diagnosed using ultrasonography by a single expert radiologist in a fasting state.

**Results:**

The mean age of the patients was 49.27 (SD 9.7) years, with 53.68% males and 46.32% females. Most patients had grade I NAFLD (72.63%), 25.26% were grade II, and 2.11% were grade III on ultrasonography. With increasing severity of liver steatosis, there were statistically significant increases in mean body mass index (P = 0.001), serum triglycerides (P = 0.026), alanine aminotransferase (P < 0.001), aspartate aminotransferase (P < 0.001), and fasting blood sugar (P = 0.041), and there was a statistically significant decrease in mean serum high-density lipoprotein cholesterol (P = 0.011). However, no association was found between severity of liver steatosis and serum total cholesterol (P = 0.271), low-density lipoprotein cholesterol (P = 0.341), and alkaline phosphatase (P = 0.234). In conclusion, the severity of ultrasonographic liver steatosis was significantly associated with abnormal metabolic parameters.

## Introduction

Non-alcoholic fatty liver disease (NAFLD) is one of the major causes of chronic liver disease worldwide and is caused by triglyceride (TG) accumulation in the liver cells of more than 5% of liver weight [1–3]. NAFLD is strongly linked to the increased prevalence of obesity, diabetes mellitus, hypertension, and hyperlipidemia [4, 5]. The disease encompasses a broad clinical spectrum of liver disorders ranging from simple steatosis to non-alcoholic steatohepatitis (NASH), fibrosis, cirrhosis, and in some cases hepatocellular carcinoma (HCC) [6]. Although liver biopsy is the gold standard method for the diagnosis of NAFLD, ultrasonography is more widely used due to increased health risks and high expenditures related to liver biopsies [4]. Therefore, NAFLD prevalence differs according to the method used for diagnosing and staging NAFLD and study population. Overall, the worldwide prevalence of NAFLD has been estimated to be 20–30% [6, 7]. However, in obese subjects, patients with type 2 diabetes mellitus, and those with metabolic syndrome, the estimated prevalence of NAFLD is much higher, ranging from 43 to 92% [8]. The prevalence of NAFLD in Asian countries has recently increased with increasing obesity, diabetes and metabolic syndrome in this region [9]. Today, NAFLD is considered as a main chronic liver disease in Asia [10]. A number of studies found that higher body mass index (BMI) was associated with an increased risk of NAFLD [3, 11–13]. Moreover, abnormal lipid metabolism or dyslipidemia is one of the common risk factors for NAFLD and is related to cardiovascular mortality, which is the most common cause of death in these patients [14–17]. Although the pathophysiology of dyslipidemia in patients with NAFLD is not fully understood, it is likely associated with hepatic overproduction of the very low-density lipoprotein particles and dysregulated clearance of different lipoproteins from the circulation [18, 19]. There are few studies investigating the relation of severity of fatty liver with metabolic disorders among Iranian population. Accordingly, this study aimed to examine the relationship between severity of liver steatosis diagnosed by ultrasound and metabolic parameters in a sample of Iranian adults.

## Main text

### Materials and methods

#### Patients

This cross-sectional study was conducted on a sample of Iranian adults referred to the gastrointestinal clinic at Imam Reza Hospital in Tabriz, Iran, from January to December 2019. The present study included 95 patients who were newly diagnosed with NAFLD. *Inclusion criteria* included subjects aged more than 20 years old and those with ultrasound-diagnosed NAFLD. *Exclusion criteria* included individuals with a history of kidney diseases, cardiovascular diseases, hypertension, diabetes, thyroid disorders, gastrointestinal disorders, those diagnosed with some pathological conditions affecting the liver such as viral hepatitis and acute or chronic liver failure, Wilson’s disease and hemochromatosis, pregnancy or breast-feeding, a history of significant alcohol intake, the use of any medication that affects lipid or glucose metabolism, adhering to a specific diet during past 3 months, and any lifestyle changes. Informed consent form was obtained from all participants before enrollment in the study. The study protocol was approved by the Ethics Committee of Tabriz University of Medical Sciences, Tabriz, Iran (ethics code; IR.TBZMED.REC.1397.694). This research is complied with the standards of the Declaration of Helsinki and current ethical guidelines.

#### Liver ultrasonography

The diagnosis of NAFLD was performed by a radiologist who was blinded to any relevant clinical information, based on ultrasonography (SonoAce X4 ultrasound system, South Korea) in a fasting state. A positive NAFLD diagnosis was attributed when ultrasound examination disclosed hepatic steatosis at any stage and in the absence of excessive consumption of alcoholic beverages (i.e., less than 20 g/day for women and less than 30 g/day for men). The following sonographic features of NAFLD were as follows: (1) normal echogenicity; (2) mild, slightly diffuse increase in bright homogenous echoes in the parenchyma, with normal visualization of the of the diaphragm and the hepatic and portal vein borders, and normal hepatorenal contrast if echogenic; (3) moderate, diffuse increase in bright echoes in the liver parenchyma, with slightly impaired visualization of the peripheral portal and hepatic vein borders; and (4) severe, marked increase in bright echoes at a shallow depth, with deep attenuation and impaired visualization of the diaphragm and marked vascular blurring.

#### Measurements

Body weights and heights were measured using a digital scale and stadiometer (Seca, Germany). BMI was calculated by dividing weight by squared height (kg/m^2^). To determine the lipid profile parameters, blood samples (5 ml) were obtained following a 12-h overnight fast. After centrifugation at 3000 rpm for 5 min, serum levels of TG, total cholesterol (TC), low-density lipoprotein cholesterol (LDL-C), high-density lipoprotein cholesterol (HDL-C), fasting blood sugar (FBS), alanine aminotransferase (ALT), aspartate aminotransferase (AST), and alkaline phosphatase (ALP) were measured by the enzymatic colorimetric method using commercial kits (Pars Azmoon Co., Tehran, Iran).

#### Statistical analysis

Statistical analysis of all data was performed using IBM SPSS Statistics software (version 23). To examine the normal distribution of variables, Kolmogorov–Smirnov tests and histograms were applied. In the present study, all variables were normally distributed. Data were presented as mean and standard deviation (SD) for continuous data and frequency (percentage) for categorical variables. To assess the correlation between severity of liver steatosis and metabolic parameters, Pearson’s correlation coefficient (r) was used. All statistical tests were two-sided, and P-values less than 0.05 were considered statistically significant.

### Results

Table 1 shows the characteristics of the study participants. The mean age of the patients was 49.27 (SD 9.7) years, with 53.68% males and 46.32% females. As shown in Table 2, most patients had grade I NAFLD (72.63%), 25.26% were grade II, and 2.11% were grade III on ultrasonography. The majority of the patients were in the age group of 50–59 (41.05%). Moreover, NAFLD was more common in the overweight (56%) or obese (class I obesity) (20%) patients. Only 14.73% of the participants had normal weight. As presented in Table 3, with increasing severity of liver steatosis, there were statistically significant increases in mean BMI (r = 0.52, P = 0.001), serum TG (r = 0.47, P = 0.026), ALT (r = 0.61, P < 0.001), AST (r = 0.52, P < 0.001), and FBS (r = 0.28, P = 0.041), and there was a statistically significant decrease in mean HDL-C (r = 0.38, P = 0.011). However, no correlation was found between severity of liver steatosis and serum TC (r = 0.14, P = 0.271), LDL-C (r = 0.22, P = 0.341), and ALP (r = 0.31, P = 0.234).

**Table 1:** Baseline characteristics of the study participants

Variable	Mean ± SD or n (%)
Age (year)	49.27 ± 9.7
Male gender	51 (53.68%)
BMI (kg/m^2^)	29.4 ± 9.7
TG (mg/dl)	166.2 ± 59.3
TC (mg/dl)	219.6 ± 74.2
LDL-C (mg/dl)	151.7 ± 66.1
HDL-C (mg/dl)	54.2 ± 19.7
FBS (mg/dl)	97 ± 29.4
ALT (U/l)	37.6 ± 16.2
AST (U/l)	27.7 ± 8.6
ALP (U/l)	188.6 ± 51.5

Data presented as the mean ± standard deviation for quantitative variable and number (%) for qualitative variables

*BMI* body mass index, *TG* triglyceride, *TC* total cholesterol, *LDL-C* low-density lipoprotein cholesterol, *HDL-C* high-density lipoprotein cholesterol, *FBS* fasting blood sugar, *ALT* alanine aminotransferase, *AST* aspartate aminotransferase, *ALP* alkaline phosphatase

**Table 2:** Degree of hepatic steatosis in different age groups

Age group (years)	Grade I—mild	Grade II—moderate	Grade III—severe
20–29	3	0	0
30–39	5	1	0
40–49	19	8	1
50–59	28	11	1
≥ 60	14	4	0
Total	69	24	2
Percentage	72.63	25.26	2.11

**Table 3:** Relationship between severity of liver steatosis and metabolic parameters

Parameters	Grade I		Grade II		Grade III		P value	r
	Mean	SD	Mean	SD	Mean	SD		
Age	51	11.7	49	9.7	44	11.4	0.651	0.11
BMI (kg/m^2^)	28.4	5.1	29.6	‏7.3	32.7	8.4	*0.001*	0.52
TG (mg/dl)	151.7	61.3	167.4	46.8	183.7	52.9	*0.026*	0.47
TC (mg/dl)	211.9	71.8	204.7	51.8	208.7	61.5	0.27	0.14
LDL-C ‏(mg/dl)	149.4	54.2	139.7	‏33.9	‏‏141.1	55.9	0.341	0.22
HDL-C (mg/dl)	58.7	24.4	‏51.1	‏16.6	42.7	18.9	*0.011*	0.38
FBS (mg/dl)	103.4	44.1	109.7	42.9	116.8	38.7	*0.041*	0.28
ALT (U/l)	32.9	‏16.8	38.8	15.2	54.9	19.9	*<* *0.001*	0.61
AST (U/l)	23.6	8.1	27.5	6.4	34.1	11.7	*<* *0.001*	0.52
ALP (U/l)	177.9	54.2	181.3	60.1	169.6	71.7	0.234	0.31

Italic values indicate significance of P value (P < 0.05)

*BMI* body mass index, *TG* triglyceride, *TC* total cholesterol, *LDL-C* low-density lipoprotein cholesterol, *HDL-C* high-density lipoprotein cholesterol, *FBS* fasting blood sugar, *ALT* alanine aminotransferase, *AST* aspartate aminotransferase, *ALP* alkaline phosphatase

P-values are based on Pearson’s correlation coefficient test

### Discussion

To the best of our knowledge, this is the first study that investigated the relationship between severity of liver steatosis diagnosed by ultrasound and metabolic parameters in Iranian adults. Our findings demonstrated that the majority of the patients had grade I NAFLD and were overweight or obese. An increasing body of evidence indicates that BMI is an independent risk factor for NAFLD [12, 13, 20, 21]. Results from a meta-analysis of 21 cohort studies demonstrated that subjects with elevated BMI had a 3.5-fold increased risk for NAFLD, and there was an obvious dose-dependent relationship between BMI and NAFLD risk [20]. In line with our results, study performed by Cuenza et al. [22] and Kim et al. [17] showed that there was statistically significant increase in mean BMI with increasing severity of NAFLD. Similarly, in the PERSIAN Guilan Cohort study (PGCS) located in the north of Iran, a significant positive relationship between BMI and risk of NAFLD was reported [11].

In the present study, severity of liver steatosis was significantly associated with serum TG, HDL-C, ALT, AST, and FBS. However, no association was found between severity of NAFLD and serum TC, LDL-C, and ALP. Similarly, a number of studies have shown a positive association between abnormal metabolic parameters and risk of NAFLD [22–25]. In line with the current study, study performed by Cuenza et al. [22] among Filipino wellness patients showed that increasing grades of NAFLD was significantly associated with high serum levels of TG, ALT, AST and FBS [23]. Moreover, significant correlation was found between severity of hepatic steatosis and increased serum levels of TC, TG, and LDL-C and decreased levels of HDL-C in the patients with NAFLD in India [24]. Furthermore, study performed by Khanal et al. [23] in adult Nepalese population demonstrated that grades of NAFLD were associated with increased serum levels of cholesterol, LDL-C, and BMI. However, no significant correlation was found between severity of NAFLD and serum levels of TG and HDL-C in the same study. In study performed by Kim et al. [17] among Korean men and women, there were statistically significant progressive increases in mean BMI, serum TC and TG, and significant decrease in mean HDL-C with increasing severity of hepatic steatosis, which support the findings of the present study. Overall, the results of this study indicate that the severity of ultrasonographic liver steatosis was significantly associated with abnormal metabolic parameters in Iranian adults.

## Limitations

The most important strength of the current study was that this study was conducted in patients newly diagnosed with NAFLD, receiving no treatment. The principal limitation of this study was the study design. Because of the cross-sectional nature of the study, one cannot infer causality. Therefore, our findings need to be confirmed in future prospective studies with larger sample sizes.

## Data Availability

The datasets used and/or analyzed during the current study are available from the corresponding author on reasonable request.

## Abbreviations

ALP: Alkaline phosphatase
ALT: Alanine aminotransferase
AST: Aspartate aminotransferase
BMI: Body mass index
FBS: Fasting blood sugar
HCC: Hepatocellular carcinoma
HDL-C: High-density lipoprotein cholesterol
LDL-C: Low-density lipoprotein cholesterol
NAFLD: Non-alcoholic fatty liver disease
TC: Total cholesterol
TG: Triglyceride
